# Outcomes Following Standardized Implantable Cardioverter-defibrillator Reprogramming

**DOI:** 10.19102/icrm.2022.130403

**Published:** 2022-04-15

**Authors:** Matthew Martini, Matthew Kalscheur, Erin Dehn, Teri McSherry, Miguel Leal, Aimee Broman, Ryan Kipp

**Affiliations:** ^1^Department of Medicine, Division of Cardiology, University of Wisconsin Hospitals and Clinics, Madison, WI, USA; ^2^Department of Medicine, Division of Cardiology, William S Middleton Veterans Affairs Hospital, Madison, WI, USA; ^3^Department of Biostatistics and Medical Informatics, University of Wisconsin-Madison, Madison, WI, USA

**Keywords:** Implantable cardioverter-defibrillator, defibrillation, device reprogramming

## Abstract

Multiple randomized controlled trials have demonstrated that programming implantable cardioverter-defibrillators (ICDs) with longer detection intervals and higher detection rates results in significant reductions in the delivery of inappropriate therapy without increasing the number of adverse events. Despite these findings, however, implementation of this evidence-based programming, particularly in previously implanted ICDs, remains inconsistent throughout the United States, with significant provider-dependent variability. We developed an institutionally standardized ICD reprogramming protocol for primary prevention ICDs utilizing high detection rates and long detection intervals, then prospectively evaluated outcomes in patients programmed with this protocol compared to a historical cohort. A total of 193 patients with primary prevention ICDs underwent standardized reprogramming and were monitored over a 1-year period. A historical cohort of 254 patients with ICD with non-standardized programming implanted prior to initiation of the standardized protocol were used as a comparison group. The primary outcomes were rates of appropriate or inappropriate ICD therapy. Secondary outcomes were rates of syncope, emergency department (ED) or urgent care (UC) visits, hospitalization, and death. All patients seen in the device clinic who qualified for device standardization were reprogrammed according to the previously developed evidence-based, institutionally standardized protocol. Patients who underwent standardized reprogramming had a lower prevalence of inappropriate therapy compared to the historical cohort (0% vs. 2.4%, *P* = .04); the prevalence of appropriate therapy was also lower in the reprogrammed group (4.1% vs. 7.1%) but not to a statistically significant degree (*P* = .19). There was a lower prevalence of syncope in the reprogrammed group (0% vs. 2.8%, *P* = .02). No significant difference in the prevalence of ED or UC utilization (37.8% vs. 33.9%, *P* = .39) or mortality (4.1% vs. 3.5%, *P* = .74) was found. Prospective standardized reprogramming of new and previously implanted primary prevention ICDs with high-rate detection and longer detection intervals may be an effective method to obtain high adherence to evidence-based reprogramming and reduce rates of inappropriate device therapies without a significant impact on appropriate therapies or mortality.

## Introduction

The use of implantable cardioverter-defibrillators (ICDs) in patients with moderate-to-severe ischemic and non-ischemic cardiomyopathy has been shown to significantly decrease mortality through the termination of ventricular arrhythmias.^1,2^ Historically, it was thought that short detection intervals and low detection rate thresholds were required to prevent sudden death from ventricular tachycardia and ventricular fibrillation. While this programming strategy increased inappropriate shocks,^3^ it was felt that rapid treatment of ventricular tachyarrhythmias was required to prevent further clinical deterioration and sudden death.

Multiple randomized controlled trials have demonstrated that programming ICDs at the time of implantation with higher detection rates and longer detection intervals can significantly reduce the rate of inappropriate therapy compared to historically conventional ICD programming.^4,5^ Despite lower rates of overall device therapies, patients programmed with higher detection cutoffs did not experience significant differences in the rates of syncope and had a lower risk of death.^5^ Previous studies have also shown that standardization of ICD programming at the time of device implantation can safely reduce inappropriate shocks.^6^

Though a large number of patients receive de novo ICD implantations each year, a much larger number of patients have devices that were implanted and originally programmed prior to the publication of the Multicenter Automatic Defibrillator Implantation Trial—Reduce Inappropriate Therapy (MADIT-RIT) and Avoid Delivering Therapies for Non-sustained Arrhythmias in ICD Patients III (ADVANCE III) trials. Despite the benefits of higher detection cutoffs and longer detection intervals, an analysis of reprogramming in practice following the publication of MADIT-RIT and a consensus document recommending high-rate or long detection programming revealed limited penetrance of recommended programming.^7^

In order to expand the benefits derived from improved programming techniques identified in the MADIT-RIT and ADVANCE III trials to all patients with ICDs, we developed an institutionally standardized protocol with a high-rate cutoff and prolonged detection time to be prospectively applied to both new and previously implanted primary prevention ICDs. We present the effectiveness of the reprogramming protocol as well as the rates of appropriate and inappropriate device therapies in the reprogrammed group compared to a historical control.

## Methods

### Study population

Reprogramming of ICDs was initially performed as a quality-improvement project to reduce the rates of inappropriate ICD therapies. All patients who were seen in the Device Clinic or implanted with a primary prevention ICD between July 1, 2016, and June 30, 2017, were included in the quality-improvement project. Patients were excluded from the study if they had a left ventricular assist device, an inherited arrhythmia, previously received appropriate ICD therapies, or ongoing use of inotropes. Outcomes in the reprogrammed group were recorded through December 31, 2017.

Patients identified as candidates for reprogramming had their devices reprogrammed using standardized parameters to allow for high-rate cutoff and prolonged detection intervals prior to ICD therapy **(Table 1)**. Patients who underwent device implantation on or after July 1, 2016, were programmed utilizing the standardized parameters at the time of implantation.

A retrospective comparison of the quality-improvement patients with a historical control was approved by the local institutional review board. Reprogrammed patients were compared against a historical cohort of primary prevention ICDs seen in the Device Clinic between January 1, 2015, and December 31, 2015. This cohort was selected to pre-date all institutionally standardized reprogramming protocols, which began on July 1, 2016. This cohort included patients who eventually underwent device reprogramming following the adoption of institutionally standardized programming parameters. Outcomes for the historical control were followed through June 30, 2016, to allow ≥6 months follow-up prior to implementation of the standardized ICD programming protocol. Any ICD therapy that occurred during this period was included with the historical control cohort. This resulted in comparable patient-years of follow-up in the historical cohort and reprogrammed groups (237.5 vs. 235.2 patient-years).

### Study endpoints

The primary endpoint was the prevalence of inappropriate ICD therapy, defined as the percentage of patients receiving any ICD therapy (anti-tachycardia pacing [ATP] or shock) delivered at any time over the study period for any rhythm other than ventricular tachycardia or ventricular fibrillation. The co-primary endpoint was prevalence of appropriate ICD therapy (ATP or shock). ICD therapies were adjudicated by 1 of 3 board-certified electrophysiologists (M. K., M. L., or R. K.). The secondary endpoints were syncope, emergency department (ED) or urgent care (UC) visits, and death as determined from a chart review.

### Statistical analysis

Baseline demographics between the historical control and the reprogrammed ICD cohort were compared using the chi-squared and Fisher’s exact tests. We compared the proportion of patients who received appropriate and inappropriate ICD therapies between the reprogrammed cohort and historical control cohort using Fisher’s exact test. The proportion of patients in each cohort with syncope, ED or UC visits, hospitalizations, or death were compared using Fisher’s exact test.

## Results

During the study period, a total of 193 patients were seen in the device clinic and qualified for device reprogramming as part of the quality-improvement project. All were reprogrammed using the standardized parameters **(Table 1)**. The historical control arm included 254 patients. There were no significant differences between the historical control and reprogrammed cohort in terms of age; sex; and rates of coronary artery disease, myocardial infarction, hypertension, or diabetes. There was no significant difference in the left ventricular ejection fraction (LVEF) **(Table 2)**.

The proportion of subjects with inappropriate therapy was higher in the historical control group compared to the reprogrammed cohort (2.4% vs. 0%; *P* = .04), while the proportion of subjects with appropriate therapies was not significantly different between the 2 study groups (7.1% in the historical control vs. 4.1% in the reprogrammed cohort, *P* = .27) **(Table 3)**.

Patients’ utilization of the ED and UC was tracked during the study period. The percentage of patients visiting the ED or UC was not different between the historical control and reprogrammed groups (33.9% vs. 37.8%, *P* = .44). There was a greater proportion of patients with reported syncope in the historical control cohort compared to the reprogrammed cohort (2.8% vs. 0%; *P* = .02). All-cause mortality between the 2 groups did not differ during the study period (3.5% vs. 4.1%; *P* = .94) **(Table 4)**.

## Discussion

In this single-center study, we found that prospectively reprogramming new and previously implanted primary prevention ICDs using a standardized protocol with higher rate-detection zones and prolonged detection times produced a high level of adherence to evidence-based programming and was associated with lower rates of inappropriate therapies compared to a historical control protocol. The proportion of patients with syncope, ED or UC utilization, or mortality was similar between the device reprogramming cohort and the historical cohort.

Previous studies, such as MADIT-RIT and ADVANCE III, have shown that device programming at the time of ICD implantation can significantly reduce the risk of inappropriate ICD therapies.^4,5^ In addition, sub-studies of MADIT-RIT have demonstrated that patients who underwent reprogramming with high-rate cutoffs were noted to have significantly better quality-of-life measurements related to their overall reduction in therapy rates.^8^

While contemporary nominal device programming facilitates adoption of high-rate detection, ICDs implanted prior to the publication of the MADIT-RIT and ADVANCE III trials most likely do not adhere to this programming standard. Despite the benefits of high-rate detection and longer detection times, investigations using the ALTITUDE registry found that, while 64% of patients with ICDs implanted prior to the publication of the MADIT-RIT trial received ICD reprogramming within 20 months of MADIT-RIT publication, only 2% were programmed to utilize the benefits of high-rate or delayed detection demonstrated in the MADIT-RIT trial with significant heterogeneity heavily dependent upon provider preference.^7^ These findings may be due to providers not wanting to adjust device programming unless an inappropriate event occurred or having previously adjusted the programming of patients with previous inappropriate or unnecessary events. However, not reprogramming an ICD to evidence-based settings unless an inappropriate event occurs may leave a large number of patients with ICDs at ongoing risk for inappropriate therapies, which may subject them to the resulting negative impacts on mental health, morbidity, and mortality.

By designing prospective standardized parameters to guide ICD programming and developing a commitment to reprograming within our device clinic, we were able to achieve consistent programming parameters for all patients with primary prevention ICDs followed in our device clinic in line with the previously published prospective trials and the Expert Consensus Statement on Optimal Implantable Cardioverter-defibrillator Programming and Testing,^9,10^ regardless of whether their ICDs were implanted before or after the publication of the ADVANCE III or MADIT-RIT trial. Given the inconsistent use of high-rate cutoffs and extended detection intervals on surveys of ICD patients nationwide, the implementation of standardized reprogramming of all new and previously implanted ICDs at an institutional level may be an effective strategy to reduce the prevalence of non-optimal device settings and, as a result, further reduce the morbidity and mortality associated with inappropriate or unnecessary device therapy.

This study had several limitations inherent in its design. First, this study was a retrospective study of a quality-improvement project that compared reprogrammed patients and a historical cohort of patients at the same institution. This design created an imbalance between the 2 arms of the study. Supraventricular tachycardia discriminators and device-detection algorithms were commonly used in the historical control group, whereas they were standardized in the reprogrammed cohort. We cannot exclude the possibility that this difference in programming also influenced the outcomes of 2 cohorts. While we were able to compare medications and comorbidities at the time of device implantation, we could not control for medication adherence or changes in comorbid conditions throughout the study period. The duration of follow-up in each cohort ranged from 6–18 months, which most likely limited our ability to detect inappropriate ICD therapy or syncope in the reprogrammed cohort. In addition, the study included primarily male veterans, limiting its generalizability to female and non-veteran populations. There are slight differences between the standardized ICD reprogramming protocol used in the study and the expert consensus statement, as this protocol was implemented before the consensus statement was published.^10^ While the differences were minor, we cannot exclude the possibility that these differences may impact the rate of ICD therapy delivery and result in different outcomes from what was seen in our study. Finally, this was a single-center study performed in the midwestern United States, which may limit generalizability of the results to other geographic areas.

## Conclusion

Standardized ICD reprogramming of patients with primary prevention ICDs seen in the device clinic created high adherence to evidence-based device programming, far exceeding the rate of adherence identified in previously published national cohorts. We found that standardized reprogramming of primary prevention ICDs to utilize high-rate cutoffs and longer detection intervals was associated with lower rates of inappropriate ICD therapy delivery without an increase in mortality, ED or UC visits, or syncope. Prospective studies should be considered to further investigate outcomes following standardized evidence-based programming of new and previously implanted ICDs.

## Figures and Tables

**Table 1:** Standardized Reprogramming for Primary Prevention Implantable Cardioverter-defibrillators

	Abbott^a^		Medtronic^a^		Boston Scientific^a^		Biotronik^a^	
Zone	Detection	Therapy	Detection	Therapy	Detection	Therapy	Detection	Therapy
VF	222 bpm (30 intervals)	ATP while charging, shock at maximum output	194 bpm (30/40 intervals)	ATP before charging, shock at maximum output	220 bpm (7-s delay)	ATP during charge, shock at maximum output	231 bpm (24/30 intervals)	ATP before charge (ATP one-shot), shock at maximum output
VT2	193 bpm (30 intervals)	ATP × 2, shock at maximum output			195 bpm (12-s delay)	ATP × 2, shock at maximum output	194 bpm (30 intervals)	ATP × 2, shock at maximum output
Monitor	150 bpm (40 intervals)		150 bpm (40 intervals)		150 bpm (20-s delay)		150 bpm (40 intervals)	

*Abbreviations:* ATP, anti-tachycardia pacing; ICD, implantable cardioverter-defibrillator; VF, ventricular fibrillation; VT, ventricular tachycardia.

^a^Abbott, Chicago, IL, USA; Medtronic, Minneapolis, MN, USA; Boston Scientific, Marlborough, MA, USA; and Biotronik, Berlin, Germany.

**Table 2:** Patient Demographics

Characteristic	Historical Control	Standardized Reprogramming	*P* value
Age (years)			
40–49	7 (2.8%)	3 (1.6%)	.08
50–59	21 (8.3%)	11 (5.7%)	
60–69	119 (46.9%)	72 (37.5%)	
70–79	83 (32.7%)	86 (44.8%)	
80–90	24 (9.4%)	20 (10.4%)	
Male (%)	250 (98.4%)	188 (97.4%)	.51
Coronary artery disease (%)	208 (81.9%)	159 (82.4%)	.99
Myocardial infarction (%)	134 (52.8%)	115 (59.6%)	.12
CABG (%)	86 (33.9%)	54 (28%)	.22
PCI (%)	102 (40.2%)	78 (40.4%)	1
Nonischemic cardiomyopathy (%)	91 (35.8%)	79 (40.9%)	.06
Atrial fibrillation or atrial flutter (%)	125 (49.2%)	91 (47.2%)	.74
Hypertension (%)	206 (81.1%)	168 (87%)	.12
Dyslipidemia (%)	227 (89.4%)	179 (92.7%)	.29
COPD (%)	75 (29.5%)	56 (29%)	.99
Diabetes (%)	114 (44.9%)	89 (46.1%)	.91
Prior or current tobacco abuse (%)	192 (75.6%)	153 (79.7%)	.23
LVEF at time of device implantation			
0%–19%	10 (4.5%)	15 (8.1%)	.47
20%–29%	125 (56.1%)	97 (52.4%)	
30%–39%	81 (36.3%)	66 (35.7%)	
40%–90%	7 (3.1%)	7 (3.8%)	
Beta-blocker (%)	209 (99.5%)	180 (99.4%)	1
ACE-I or ARB (%)	194 (92.4%)	169 (92.9%)	1
Amiodarone (%)	10 (4.8%)	8 (4.4%)	1
Sotalol (%)	1 (0.5%)	2 (1.1%)	.60
Device manufacturer			
Medtronic	129 (50.8%)	125 (64.8%)	.03
Boston Scientific	70 (27.6%)	38 (19.7%)	
Abbott	47 (18.5%)	23 (11.9%)	
Biotronik	6 (2.4%)	5 (2.6%)	
Other	2 (0.8%)	2 (1%)	

*Abbreviations:* ACE-I, angiotensin converting enzyme-inhibitor; ARB, angiotensin receptor blocker; CABG, coronary artery bypass grafting; COPD, chronic obstructive pulmonary disease; LVEF, left ventricular ejection fraction; PCI, percutaneous coronary intervention.

**Table 3:** Rate of Appropriate and Inappropriate Implantable Cardioverter-defibrillator Therapy in the Historical Control and Standardized Reprogramming Cohorts

	Historical Control	Standardized Reprogramming	*P* value
**Appropriate therapy**	**18 (7.1%)**	**8 (4.1%)**	**.27**
Appropriate ATP	12 (4.7%)	5 (2.6%)	.36
Appropriate shocks	5 (2%)	1 (0.5%)	.24
Appropriate ATP and shock	1 (0.4%)	2 (1.0%)	1.00
**Inappropriate therapy**	**6 (2.4%)**	**0 (0%)**	**.04**
Inappropriate ATP	2 (0.8%)	0 (0%)	.51
Inappropriate shock	0 (0%)	0 (0%)	1.00
Inappropriate ATP and shock	4 (1.6%)	0 (0%)	.14

*Abbreviation:* ATP, anti-tachycardia pacing.

**Table 4:** Rate of Emergency Department or Urgent Care Visits, Syncope, and Mortality in the Historical Control and Standardized Reprogramming Cohorts

	Historical Control	Standardized Reprogramming	*P* value
Any ED or UC visit	86 (33.9%)	73 (37.8%)	.44
Syncope	7 (2.8%)	0 (0%)	.02
Mortality	9 (3.5%)	8 (4.1%)	.94

*Abbreviations:* ED, emergency department; UC, urgent care.
